# Assessing the extent to which current clinical research is consistent with patient priorities: a scoping review using a case study in patients on or nearing dialysis

**DOI:** 10.1186/s40697-015-0070-9

**Published:** 2015-10-01

**Authors:** Min Jun, Braden Manns, Andreas Laupacis, Liam Manns, Bhavdeep Rehal, Sally Crowe, Brenda R. Hemmelgarn

**Affiliations:** University of Calgary, Cumming School of Medicine, Division of Nephrology, Health Sciences Building, 3330 Hospital Drive NW, Calgary, T2N 4N1 AB Canada; Department of Community Health Sciences, University of Calgary, Calgary, AB Canada; Li Ka Shing Knowledge Institute of St Michael’s Hospital, Toronto, ON Canada; Faculty of Medicine, University of Toronto, Toronto, ON Canada; Oxford, UK

## Abstract

**Purpose of review:**

There is growing acknowledgement that engaging patients to identify their research priorities is important. Using a case study of patients on or nearing dialysis, we sought to assess the extent to which recently completed and ongoing clinical research was consistent with priorities identified by patients, caregivers, and clinicians.

**Sources of information:**

Over a 4-year sampling frame (January 2010 to December 2013), we systematically searched the medical literature (top 5 nephrology and top 10 general medicine journals accessed through MEDLINE via Ovid), international randomized controlled trial (RCT) registries, and national government and kidney research funding organizations (Canada, U.S., Australia, and U.K.) for published clinical studies, registered RCTs, and funded clinical studies, respectively. Published clinical studies, registered RCTs, and funded clinical studies were categorized as to whether or not they were consistent with the top 10 research priorities identified by patients, their caregivers, and clinicians in a recent comprehensive research priority setting exercise.

**Findings:**

The search yielded 4293 published articles, 688 RCTs, and 70 funded studies, of which 1116 articles, 315 RCTs, and 70 funded studies were eligible for inclusion. Overall 194 published studies (17.4 %), 71 RCTs (22.5 %), and 15 funded studies (21.4 %) included topics consistent with the top 10 research priorities identified by patients. Four of the top 10 research priorities, including strategies to improve the management of itching, increase access to kidney transplantation, assess the psychosocial impact of kidney failure, and determine the effects of dietary restriction received virtually no attention.

**Limitations:**

The top 10 priorities we used to categorize included studies were identified by Canadian patients, caregivers, and clinicians. The top research priorities may vary across different countries. The proportion of published studies that are consistent with the top 10 priorities could be different in nephrology journals with lower impact factors. Studies related to kidney transplantation and the psychosocial impact of kidney failure may have been published in journals not included in our search strategy.

**Implications:**

The majority of recently completed or ongoing clinical studies in patients on or nearing dialysis do not address the top research priorities of patients, raising concerns that current clinical research may not be meeting the needs of the ultimate consumer, in this case, patients on or nearing dialysis. Greater involvement of patients in research is required to bridge the gap between research and patients’ needs.

**Electronic supplementary material:**

The online version of this article (doi:10.1186/s40697-015-0070-9) contains supplementary material, which is available to authorized users.

## Abrégé

**Objectifs de l’étude:**

Il apparait de plus en plus important de consulter les patients afin de mieux répondre à leurs attentes vis-à-vis de la recherche clinique. Lors d’une étude de cas faite sur des patients en dialyse ou sur le point d’en entreprendre une, nous avons voulu savoir à quel point les études cliniques en cours ou complétées récemment étaient en accord avec les priorités de recherche établies par les patients, le personnel soignant et le personnel clinique.

**Sources:**

Pour ce faire, nous avons dressé un portrait de la situation sur une période de quatre ans (de janvier 2010 à décembre 2013). Nous avons passé en revue de façon systématique la littérature médicale (les cinq meilleures revues scientifiques en néphrologie et les dix journaux de médecine générale les plus cités accessibles sur MEDLINE via Orbid) ainsi que les registres internationaux d’essais cliniques. Nous avons répertorié les études cliniques publiées, les essais cliniques déposés et les études cliniques financées au privé parmi les publications gouvernementales à l’international (Canada, États-Unis, Australie et Royaume-Uni) et les rapports des organismes finançant la recherche sur les maladies du rein. Nous voulions évaluer la cohérence entre les objectifs et résultats de ces recherches et les dix principales attentes des patients, du personnel soignant et du personnel clinique face à celles-ci.

**Constatations:**

La recherche a mené à une liste de 4293 articles publiés, 688 essais cliniques et 70 études cliniques financées, de laquelle 1116 articles, 315 essais cliniques et 70 études cliniques financées étaient admissibles à satisfaire nos critères de recherche. Finalement, seulement 194 articles publiés (17.4 %), 71 essais cliniques (22.5 %) et 15 études financées (21.4 %) mentionnaient l’une ou l’autre des dix priorités de recherche établies par les patients, le personnel soignant ou le personnel clinique. Quatre de leurs préoccupations principales, soit la façon d’améliorer le traitement des démangeaisons, l’accès accru aux transplantations rénales, l’évaluation de l’impact de l’insuffisance rénale au point de vue psychosocial chez le patient atteint ainsi que la détermination des risques potentiels posés par les restrictions alimentaires sévères, n’ont reçu que très peu d’attention parmi les sources passées en revue.

**Limites de l’étude:**

La liste des dix priorités de recherche utilisée pour classer l’information recueillie parmi les sources citées avait été établie par des patients, du personnel soignant et du personnel clinique canadien seulement. Ces priorités pourraient varier d’un pays à l’autre. Aussi, le passage en revue des journaux de néphrologie de moindre importance aurait possiblement mené à des résultats différents. Il est tout à fait possible que des études sur l’impact au point de vue psychosocial de l’insuffisance rénale aient été publiées dans des journaux n’ayant pas fait partie de notre stratégie de recherche.

**Conséquences:**

Il ressort de cela que la majorité des études cliniques en cours ou complétées dans les dernières années ayant été faites sur des patients en dialyse ou sur le point d’en entreprendre une n’abordait pas les principales attentes des patients. Ce fait est préoccupant puisqu’il implique que la recherche actuelle ne répond pas adéquatement aux besoins des gens à qui elle s’adresse ultimement. Par conséquent, l’implication plus directe des patients dans la recherche est requise pour établir des ponts et réduire l’écart entre celle-ci et les besoins réels des usagers.

## What was known before

There is increasing recognition of the importance of conducting patient-oriented research, that is, research which focuses on the priorities and outcomes relevant to patients, as indicated by the establishment of national initiatives such as the Strategy for Patient Oriented Research (SPOR) in Canada and the Patient-Centered Outcomes Research Institute (PCORI) in the United States.

## What this adds

We found that approximately only 1 in 5 current clinical research in dialysis addressed 1 of the top 10 research priorities identified by dialysis patients, their caregivers, and clinicians, highlighting the critical gap between current research in dialysis and what dialysis patients consider to be high priority.

## Background

There is increasing recognition of the importance of patient-oriented research, defined as the design and conduct of research that respects and focuses on the priorities and outcomes relevant to patients [1–3]. It is felt that by addressing the needs of those who are most impacted by their disease, research will be better able to inform clinicians, health systems managers, policy makers, and patients themselves about the most appropriate and effective care.

Although there is increasing focus on the importance of conducting research relevant to patients in Canada (Strategy for Patient-Oriented Research [SPOR]), the US (Patient-Centered Outcomes Research Institute [PCORI]) and elsewhere, the proportion of funding currently directed towards patient-oriented research is modest. [4] Given this and the fact that patients and their caregivers have not traditionally been well represented in health research and priority-setting activities [1, 5, 6], current research may not address the most important areas of concern for patients and their caregivers. Evidence about the extent of this potential disparity is limited and dated, but suggests that there may be discrepancies between current research and patient priorities [7].

Recently, the top 10 research priorities of Canadian patients on or nearing dialysis, and the caregivers and clinicians who care for them, were identified [8] using a method developed by the James Lind Alliance, which brings together patients, caregivers and clinicians to identify the most important uncertainties for research [9]. Based on this case study of patients on or nearing dialysis, the objective of this study was to determine the extent to which research recently published in high impact general medicine and nephrology journals and ongoing clinical research is consistent with the top priorities of patients.

## Methods

### Data sources and search strategy

We conducted a scoping review [10], based on a protocol developed *a priori*, to systematically identify clinical research studies published, registered, or funded that focused on adult patients on or nearing dialysis between January 1, 2010 and December 31, 2013 from 3 sources based on relevant medical subject headings and text words (Table 1; see Additional file 1 for search strategy). For published clinical research studies (irrespective of intervention, comparator, outcome, or study type), we examined the top 10 general medicine and top 5 nephrology journals (based on impact factors from the ISI Web of Knowledge Journal Citation Reports) accessed through MEDLINE via Ovid (2010–2013). To assess registered randomized controlled trials (RCTs; irrespective of intervention, comparator, or outcome), we examined the World Health Organization International Clinical Trials Registry Platform which indexes RCT registries of the U.S., U.K, countries of the European Union, Africa, and 11 countries in Asia, South America, and the Caribbean (http://apps.who.int/trialsearch/). An RCT was defined as a study involving the random allocation of participants to treatment arms or a study using a cross-over design. To assess recently funded studies (irrespective of intervention, comparator, outcome, or study type), we examined publicly searchable websites of national government and kidney research funding organizations of 4 countries: Canada, U.S., Australia, and U.K. Links to the websites are provided in Additional file 1. All searches were without language restriction.

**Table 1:** Summary of the search strategy and the inclusion and exclusion criteria for the review

	Published studies	Registered trials	Funded studies
Sources searched	Top 10 ranked general medicine and top 5 ranked nephrology journals	WHO International Clinical Trial Registry Platform	National and kidney research funding organizations of Canada, USA, Australia, and UK
Study period	2010 – 2013		
Inclusion criteria	Clinical investigations^a^ including adults (≥ 18 years of age) on or nearing dialysis (HD, PD, or both)		
Exclusion criteria	Studies in basic science (pre-clinical)		
	Studies which included only patients with earlier stages of CKD		

^a^A clinical investigation was defined as a study assessing disease prevention, management, and other studies related to the impact of healthcare systems on patient outcomes; *HD* hemodialysis, *PD* peritoneal dialysis; *CKD* chronic kidney disease

### Study selection

Two reviewers independently screened abstracts of published articles, registered RCTs, and funded studies for eligibility. Studies were deemed eligible and included in the review if they were original clinical investigations including adult (≥18 years) patients on or nearing chronic dialysis (hemodialysis, peritoneal dialysis, or both). A clinical investigation was defined as a study assessing disease prevention, management (including detection, diagnosis, prognosis, and assessment of treatments), and other studies related to the impact of healthcare systems on outcomes. Clinical investigations included translational research, defined as studies based on the collection of human blood or tissue samples to assess biomarkers or imaging technologies. Studies in basic science, defined as pre-clinical laboratory-based studies (e.g. animal models) concerned with understanding the basic mechanisms of disease (biological or physiological) were excluded. For the search of studies funded by research funding organizations, we included only operating grants that supported the conduct of research; grants supporting individuals such as fellowship awards, were excluded. Studies conducted in kidney transplant recipients were excluded; however, studies aimed at strategies to increase kidney transplantation (e.g. access to kidney transplantation) were included. Studies which included only patients with earlier stages of CKD (defined as patients being >1 year from expected dialysis) were excluded. Any disagreements in the inclusion of studies were adjudicated by a third reviewer.

### Data extraction

Data from eligible studies were abstracted independently by 2 reviewers using standardized spreadsheets; discrepancies in the extracted data were reviewed and resolved by a third reviewer. The data abstracted included the year the study was published, registered, or funded, the type of dialysis (pre-dialysis, hemodialysis, peritoneal dialysis, or both dialysis modalities), study design (RCT or observational), the funding type (academic [including public funding] or commercial), and the total funded amount (for studies identified through national and kidney research funding organizations). We also determined whether relevant studies assessed outcomes of importance to patients as their primary outcome of interest– defined as all-cause mortality, myocardial infarction, stroke, heart failure, and quality of life [assessed by a range of surveys or instruments as defined by authors]). Surrogate outcomes such as biochemical markers (e.g. iron and phosphate) were not considered clinically important outcomes for patients.

### Data synthesis

#### The top 10 dialysis research priorities

The top 10 dialysis research priorities were identified through a survey of Canadian adult patients on or nearing dialysis, their caregivers, and health care professionals (total of 317 including 173 patients, 107 health care professionals, and 37 caregivers), followed by an in-person workshop of 34 people based on the James Lind Alliance method [9]. The top 10 research priorities included topics related to determining ways to enhance communication among patients and care providers, dialysis modality options, management of itching, access to kidney transplantation, psychosocial impact of kidney failure, cardiovascular health, dietary restrictions, management of depression, management of complications arising from dialysis, and vascular access (Table 2). Details about the methodology used is provided elsewhere [8].

**Table 2:** The top 10 dialysis research priorities [8]

Top 10 dialysis research priorities identified by dialysis patients, their carers, and clinicians
1. What is the best way of informing patients with kidney failure about the advantages and disadvantages of different forms of dialysis; and how can we ensure that people get the right information, at the right time, and in the right way to ensure informed decision-making?; How can communication between patients with kidney failure and health care providers be improved, and does enhanced communication (including providing test results) increase patients’ ability to participate in the management of their condition?
2. How do the different dialysis modalities compare with one another in terms of their impact on quality of life and mortality, and are there specific patient factors that make one modality better for some patients with kidney failure than others?; How can hemodialysis be tailored to a patient [in terms of: length, frequency, location and schedule (e.g. day/night-time)] to enhance effectiveness and quality of life?
3. What are the causes and effective treatment(s) of, and ways to prevent, itching in dialysis patients?
4. What is the best strategy to increase kidney transplantation; including access to transplantation, increasing the efficiency of the recipient workup, and increasing the availability of donor kidneys?
5. What is the psychological and social impact of kidney failure on patients, their family, and other caregivers, and can this be reduced?
6. What are the best ways to promote heart health in dialysis patients, including management of blood pressure?
7. For people with kidney failure, what is the impact of each of the dietary restrictions (sodium, potassium, phosphate) separately, and when taken in combination, on important outcomes including quality of life?
8. What are the best ways to manage or prevent complications that occur during or shortly after the hemodialysis treatment itself (i.e. low blood pressure, cramping, nausea, headaches)?
9. What are the causes and effective treatment(s) of depression in dialysis patients?
10. What is the best type of access (among both new and existing varieties) for people on hemodialysis?

#### Categorization of studies

Two reviewers independently allocated the eligible studies into 1 of the 10 research priorities identified by patients and caregivers. If the study did not match any of the top 10 priorities, it was categorized as not being consistent with the top 10 priorities. Any disagreements were resolved by discussion and consensus. Agreement between the 2 reviewers regarding the categorization of abstracts according to the top 10 priorities was calculated using the kappa statistic [11]. As it was also deemed important to describe the studies that did not address the top 10 priorities, studies which did not match at least 1 of the top 10 priorities were categorized using a taxonomy of kidney research topics established by the American Journal of Kidney Diseases (AJKD), which includes 41 major research topics in kidney disease. This taxonomy includes acute kidney injury (AKI) as a research area but because the identification of the top 10 research priorities was focused on chronic dialysis patients, we excluded all studies assessing acute dialysis in patients with AKI.

Seven of the top 10 research priorities identified by patients and caregivers (determining ways to enhance communication among patients and care providers, dialysis modality options, access to kidney transplantation, cardiovascular health, dietary restrictions, management of complications arising from dialysis, and vascular access) overlap with 7 of the 41 research topic areas identified by AJKD (“Cardiovascular disease”, “Hemodialysis”, “Nutrition”, “Hypertension”, “Peritoneal dialysis”, “Quality of life”, and “Transplantation”). In some cases, recently published papers, registered RCTs, or currently funded research were focused on 1 of the patient priorities (e.g. effect of beta-blockers on cardiovascular outcomes); in these cases the studies were classified as addressing 1 of the patient-identified research priorities. In other cases (e.g. a study investigating for an association between cardiovascular disease and cognitive function in hemodialysis patients), even though the research was related to a broad topic identified by patients (e.g. heart health), it was felt not to be focused on 1 of the patient priorities and was not classified as addressing 1 of the patient-identified research priorities.

The proportion of eligible studies that addressed each of the top 10 priorities across the published medical literature, international RCT registers, and national and kidney research funding organizations was determined and displayed graphically. Descriptive statistics were performed using Stata version 11 (StataCorp, College Station, Texas).

## Results

### Search results and characteristics of the included studies

**Fig. 1:**
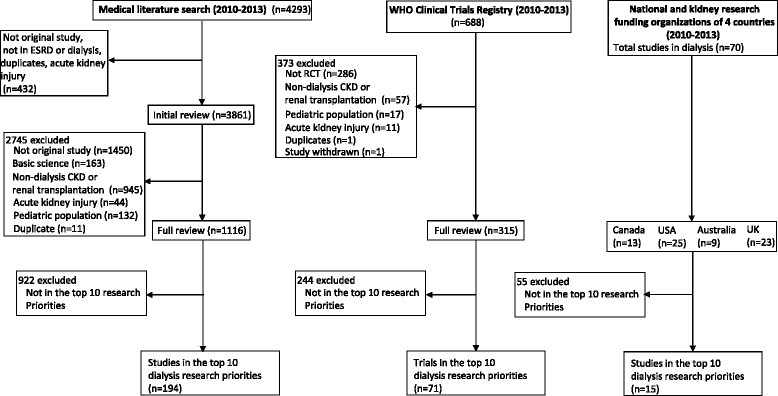
Identification of eligible studies

Our initial search yielded 4293 published articles from the medical literature and 688 RCTs in the international RCT registries. Of these, 1116 articles and 315 RCTs were eligible for inclusion in our review. Studies were excluded because they were not original investigations (e.g. reviews), included patients with earlier stages of CKD not requiring dialysis, included critically ill patients with AKI, or because they were duplicates of studies already identified (Fig. 1). The search of major national and kidney research funding organizations in Canada, U.S., Australia, and U.K. yielded 70 eligible studies. Among the eligible studies identified from the medical literature, international RCT registries, and national and kidney research funding organizations, the majority of studies assessed chronic hemodialysis exclusively (55.7, 70.5 and 48.5 %, respectively; Table 3). The majority of studies identified from the medical literature and funding organizations were observational studies (87.9 and 90 %, respectively). Only a minority of identified studies included patient-relevant clinical outcomes as the primary outcome of the study (30.9, 15.9, and 32.9 %, respectively).

**Table 3:** Characteristics of the identified studies including adult patients on or nearing chronic dialysis

	Published studies in the medical literature	Registered RCTs	Studies identified through funding organizations of Canada, U.S., Australia, and U.K.
Study designs included	RCTs and observational studies	RCTs	RCTs and observational studies
Eligible studies; n	1116	315	70
Studies in the top 10 priorities; n (%)	194 (17.4)	71 (22.5)	15 (21.4)
Study type (%)			
RCT	12.1	100	10
Observational	87.9	0	90
Dialysis status (%)			
Hemodialysis	55.7	70.5	48.5
Peritoneal dialysis	12.0	19.4	15.7
Both	29.9	10.2	35.7
Nearing dialysis	2.3	0	0
Assessed clinical outcomes as part of the primary outcome of the study (%)	30.9	15.9	32.9
Primary funding type (%)			
Academic^a^	40.6	79.3	100
Commercial^b^	13.3	20.3	0
Unspecified	46.2	0.3	0

*RCT* randomized controlled trial, ^a^Including public and not-for-profit funding; ^b^including studies from the pharmaceutical industry

### The extent to which published or ongoing studies addressed the top 10 research priorities

**Fig. 2:**
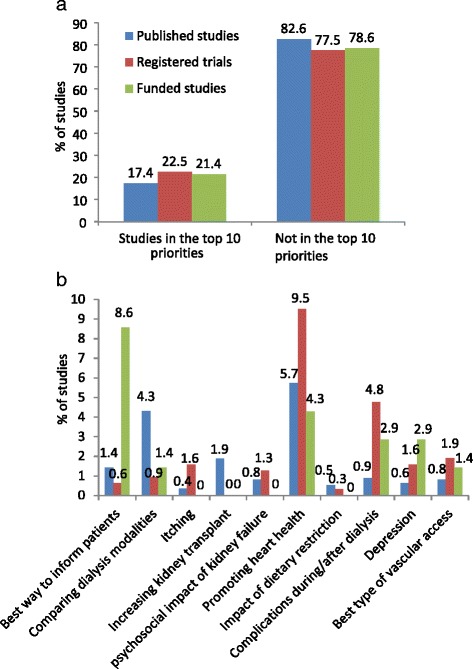
**a** The proportion of eligible studies, by information source (published literature, RCT registry, and funding organizations), identified as addressing one of the top 10 research priorities compared to studies which did not assess one of the top 10 priorities, **b** Distribution of studies addressing one of the top 10 research priorities, by the top 10 dialysis patient priorities

There was good agreement between the 2 reviewers for the categorization of published studies, registered RCTs, and studies identified through funding agencies according to the top 10 priorities (Kappa = 0.82, 0.97, 0.88, respectively). The proportion of studies that addressed the top 10 priorities was 17.4 % in the medical literature, 22.5 % in the RCT registries, and 21.4 % in the funding organizations (Table 3 and Fig. 2a). Among studies addressing 1 of the top 10 research priorities, strategies aimed at improving cardiovascular health in dialysis patients were the most common (Fig. 2b). Across the 3 sources, very few studies focused on itching, increasing access to kidney transplantation, assessing the effect of dietary restriction, or describing the psychosocial impact of kidney failure; none of the 70 studies supported by the funding organizations addressed these issues.

Overall, the proportion of studies consistent with the top 10 dialysis patient priorities was similar across the years from 2010 to 2013 (Additional file 2).

### The proportion of recent research funding directed to studies addressing the top 10 research priorities

Of the 70 eligible clinical research studies identified through national and kidney research funding organizations (funded during 2010–2013), 13 were in Canada (total amount funded: CA $844,158), 25 were in the U.S. (US $9,342,407), 9 were in Australia (AU $3,229,756), and 23 in the U.K. (UK ₤3,503,347). The proportion of research funding provided to studies addressing 1 of the top 10 dialysis research priorities across the 4 countries was 22.3, 20.6, 33.3, and 26.3 % respectively.

### The areas of research among studies not addressing the top 10 dialysis research priorities

Additional file 3 describes the areas of focus of the studies that did not address the top 10 research priorities; assessment of hemodialysis-related interventions (unrelated to the top 10 research priorities (e.g. effect of acetate-free hemodiafiltration on cardiac troponin levels) was the most common topic across the medical literature, international RCT registries, and national and kidney research funding organizations (16.9, 29.1, and 36.4 % respectively).

## Discussion

We found that most currently conducted clinical research does not address the top 10 research priorities identified by patients and their caregivers. Only about 20 % of clinical research, whether we looked at the medical literature published in the top 10 general medicine and top 5 nephrology journals, international RCT registries, or major national funding organizations, addressed the top 10 priorities relevant to patients on or nearing dialysis.

Even among studies that did focus on the top 10 patient priorities, there was wide variation in the attention received by each of the 10 priorities with 4 of them receiving virtually no attention. This is particularly concerning as most of these 4 priorities have been consistently identified as important areas by patients [12–14].

The ultimate objective of nearly all health research is to improve the health outcomes of patients. Our findings show a critical gap between current research and what patients considered to be high priority, and may reflect the lack of patient engagement in research priority setting [15, 16]. There is a limited number of studies comparing the research priorities of patients and healthcare providers [17]. Our results are consistent with a previous study which focused on interventions for the treatment of osteoarthritis but that study did not use an established methodology to elicit patient priorities and was published over a decade ago [7]. Similar discrepancies have been observed across other areas of medicine [18, 19].

### Why is there a gap between current research and the priorities of patients and why is this a problem?

Despite reports of increasing consumer involvement in research as early as 2001 [20], there remains a large disparity between current research and the priorities of patients. This persistent gap may be explained by the lack of engagement of patients over the entire breadth of research [14] and particularly by the limited involvement of patients during research proposal-setting processes [21] which have largely been driven by researchers often based on their own interests, the burden of disease [4, 22] as well as commercial interests. This is concerning because it suggests that the perspectives of those who may be most affected by the research evidence (i.e. patients) is not being incorporated in the conduct of current research. Consequently, the needs of patients may not be sufficiently met by funding agencies which are largely supported by public funds. We believe that addressing this gap is important for several reasons. First and foremost is the concept of fairness and justice - patients and their caregivers are the individuals most impacted by their disease, and they should be able to contribute to establishing research priorities. Indeed when provided with the opportunity, patients are not only capable of contributing to research agenda prioritizations but can also significantly change it. A recent cluster RCT assessing the impact of involving patients in setting priorities for healthcare improvement in chronic care, noted that the priorities established through the incorporation of patients were significantly different than those set by healthcare professionals alone [23].

Translating research findings into clinical practice has been challenging [24]. The incorporation of patient perspectives in research will likely improve the translation of research results into clinical practice and policy by answering questions of highest relevance to patients and their caregivers [25]. In addition, patients and the public may be more likely to support the funding of research if they view the research as relevant, ultimately allowing for better implementation of study findings.

### How do we bridge the gap between current research and the priorities of patients?

While the overall gap between current research and patient priorities in this study was large, there is increasing awareness of the importance of patient priorities and some granting agencies are developing strategies to increase their focus on patient-oriented research. The U.K. has been active in this field and has been formally involving patient perspectives in research for almost 2 decades through INVOLVE, a National Institutes of Health Research (NIHR) initiative established in 1996 to support active public involvement in NIHR research [26]. The U.S. has established PCORI in 2010, which will receive $3.5 billion through to September 2019 to determine effective strategies to improve the outcomes of patients through patient involvement [27]. In Canada, SPOR was established in 2011 to focus on interventions and outcomes considered important by patients [1].

Better engagement of patients and their caregivers needs to be achieved to increase incorporation of their perspectives in research. Both INVOLVE and PCORI recommend the involvement of patients through the continuum of research, including the formulation of the research question, study conduct, (e.g. development of patient-tailored survey tools), and the dissemination of results (e.g. identify dissemination methods to maximize exposure) [28–30]. Reports from INVOLVE assessing the impact of patient involvement on research identified a number of benefits including the development and clarification of the research question resulting in a better understanding of the needs of patients and overall improvements in recruitment rates and data collection [31, 32]. The James Lind Alliance research priority setting process supports the use of surveys as well as face-to-face interviews to form priority-setting partnerships which bring together patients, their caregivers, and clinicians to elicit research uncertainties [33] which have been implemented across varying fields of medicine [8, 33–36].

We are not suggesting that all research must focus on patient priorities. Investigator-initiated research, particularly in the basic sciences, allows for innovative and sometimes unanticipated discoveries, generating new ideas and findings which have important implications for the management of patients in the future. This review does not negate the importance of other areas of research beyond the patient priorities (or research in basic science) but rather reinforces the need to better incorporate the perspectives of patients and their carers, perhaps through targeted funding strategies, so that the gap between current research and the priorities of patients can be narrowed.

Our review was based on a systematic and comprehensive search strategy to identify the most recently completed and ongoing studies from multiple international sources. However, our study has limitations. The top 10 research priorities of patients and caregivers were determined by a Canadian survey and workshop, and it is likely that another priority setting exercise would have yielded a slightly different top 10. However, we used a standard methodology [9] and are confident that the top 10 priorities are all important to patients and their caregivers. In addition, while the top 10 priorities we used to categorize included studies were identified through a Canadian survey, the published literature, RCT registries, and funding organizations were not limited to Canada. The top research priorities may vary across different countries. It is possible that certain published studies consistent with the top 10 priorities (e.g. those related to kidney transplantation and the psychosocial impact of kidney failure) were published in journals not included in our search strategy. It is also possible that the proportion of papers that are consistent with patient priorities is different in journals with lower impact factors. While the priorities identified by patients could in some cases be addressed by basic science experiments, our review excluded studies in these areas because basic science research is often directed toward understanding basic disease mechanisms, and it is difficult to determine their relevance to patients for some time. This makes it challenging to categorize many basic science research studies according to the top 10 dialysis research priorities. We included all clinical investigations, including translational research (defined in Methods), where the intent and relevance of the study to the top 10 categories was easier to assess. Finally, our research priority setting process did not involve policymakers and it is possible that their priorities would have been different from the priorities identified by patients and their caregivers.

## Conclusion

The majority of current research in patients on or nearing dialysis is in areas outside the top research priorities identified by patients, their caregivers, and their health care providers. Greater involvement of patients and their caregivers is needed so that the disparity between research and the needs of patients can be bridged.

## Additional files

Additional file 1:
**Search strategy.** (DOCX 22 kb) Additional file 2:
**The distribution of the proportion of studies which were consistent with at least 1 of the top 10 dialysis patient priorities by year (2010 – 2013).** (DOCX 81 kb) Additional file 3:
**Studies not addressing the top 10 research priorities categorized based on a 41 category taxonomy of major kidney research areas.** (DOCX 69 kb)
