# Supplementary material for: Assessing the extent to which current clinical research is consistent with patient priorities: a scoping review using a case study in patients on or nearing dialysis
Source: Can J Kidney Health Dis. 2015 Oct 1;2:35. doi: 10.1186/s40697-015-0070-9 (PMC4590701; doi:10.1186/s40697-015-0070-9)
Supplement: Additional file 1: — Search strategy. (DOCX 22 kb) [file 40697_2015_70_MOESM1_ESM.docx]

**Additional file 1**

Published studies in the medical literature (2010-2013)

Top-5 nephrology journals (by impact factor as of 2012 – ISI Web of Knowledge Journal Citation Reports)

1. Journal of the American Society of Nephrology (JASN)
2. Kidney International (KI)
3. American Journal of Kidney Diseases (AJKD)
4. Clinical Journal of the American Journal of Nephrology (CJASN)
5. Nephrology, Dialysis, Transplantation (NDT)

Top-10 general medicine journals (by impact factor as of 2012 – ISI Web of Knowledge Journal Citation Reports)

1. New England Journal of Medicine (NEJM)
2. Lancet
3. Journal of the American Medical Association (JAMA)
4. British Medical Journal (BMJ)
5. Public Library of Science Medicine (PLoS Medicine)
6. Annals of Internal Medicine
7. JAMA Internal Medicine (previously known as Archives of Internal Medicine)
8. Biomed Central Medicine (BMC Medicine)
9. Canadian Medical Association Journal (CMAJ)
10. Journal of Internal Medicine

Registered trials – World Health Organization (WHO) International Clinical Trials Registry Platform (http://apps.who.int/trialsearch; 2010-2013)

The WHO Clinical Trials Registry indexes the following national trial registries:

1. ClinicalTrials.gov
2. Australia New Zealand Clinical Trials Registry
3. EU Clinical Trials Register
4. International Standard Randomised Controlled Trial Number Register
5. Brazilian Clinical Trials Registry
6. Chinese Clinical Trial Registry
7. Clinical Trials Registry – India
8. Clinical Research Information Service – Republic of Korea
9. Cuban Public Registry of Clinical Trials
10. German Clinical Trials Register
11. Iranian Registry of Clinical Trials
12. Japan Primary Registries Network
13. Pan African Clinical Trials Registry
14. Sri Lanka Clinical Trials Registry
15. The Netherlands National Trial Register
16. Thai Clinical Trials Register

National funding agencies and kidney research funding organisations of Australia, Canada, U.K., and U.S. (2010-2013)

1. Australia
   1. National Health and Medical Research Council (NHMRC); <https://www.nhmrc.gov.au/grants/research-funding-statistics-and-data>
   2. Kidney Health Australia (KHA) & Australia and New Zealand Society of Nephrology (ANZSN); <http://www.kidney.org.au/HealthProfessionals/MedicalResearchFunding/tabid/633/Default.aspx>
   3. Heart Foundation; <http://www.heartfoundation.org.au/research/current-research/Pages/welcome.aspx>
   4. National Stroke Foundation; <http://strokefoundation.com.au/research/research-grants/>
   5. Diabetes Australia; <http://www.diabetesaustralia.com.au/Research/DART/>
2. Canada
   1. Canadian Institutes of Health Research (CIHR); <http://www.cihr-irsc.gc.ca/e/196.html>
   2. Kidney Foundation of Canada (KFOC) & Canadian Society of Nephrology (CSN); <http://www.kidney.ca/page.aspx?pid=353>
   3. Heart and Stroke Foundation; <http://www.hsf.ca/research/en/20142015-grant-aid-grantees>
   4. Canadian Diabetes Association; <http://www.diabetes.ca/research/research-grants-awards-competitions>
3. UK
   1. National Institutes of Health Research (NIHR); <http://www.ccf.nihr.ac.uk/RfPB/about/Pages/FundedProjects1.aspx> ; <http://europepmc.org/GrantLookup/>
   2. Medical Research Council (MRC); <http://europepmc.org/GrantLookup/>
   3. Kidney Research UK; <http://www.kidneyresearchuk.org/research>
   4. British Renal Society; <http://www.britishrenal.org/Research-for-Renal/Grants-Awarded.aspx>
   5. British Heart Foundation; <http://www.bhf.org.uk/research/research-grants/previous-awards.aspx?sc_id=FP-00282&utm_source=SLP-Re-Grants-1&utm_medium=SLP-Re-Grants-1&utm_campaign=SLP-Re-Grants-1%20071210>
   6. Stroke Association; <http://www.stroke.org.uk/research/search>
   7. Diabetes UK; <http://www.diabetes.org.uk/Research/Our-research-projects/>
4. USA
   1. National Institutes of Health (NIH); <http://report.nih.gov/index.aspx>
   2. National Kidney Foundation; <http://www.kidney.org/professionals/research/awards.cfm>
   3. American Heart Association; <http://my.americanheart.org/professional/Research/Research_UCM_316889_SubHomePage.jsp>
   4. American Stroke Association; <http://my.americanheart.org/professional/Research/Research_UCM_316889_SubHomePage.jsp>
   5. American Diabetes Association; <http://www.diabetes.org/research-and-practice/we-are-research-leaders/>

Search strategy for studies in the medical literature (MEDLINE via Ovid; 2010-2013)

1. Journal of the American Society of Nephrology.jn
2. Kidney International.jn
3. American Journal of Kidney Diseases.jn
4. Clinical Journal of the American Society of Nephrology cjasn.jn
5. Nephrology, Dialysis, Transplantation.jn
6. New England Journal of Medicine.jn
7. Lancet.jn
8. Journal of the American Medical Association.jn
9. British Medical Journal.jn
10. PLOS Medicine public library of science.jn
11. Annals of Internal Medicine.jn
12. Archives of Internal Medicine.jn
13. JAMA Internal Medicine.jn
14. BMC Medicine.jn
15. Canadian Medical Association Journal.jn
16. Journal of Internal Medicine.jn
17. Or/1-16
18. limit 17 to yr="2010 - 2013"
19. exp Renal Insufficiency, Chronic/
20. exp Renal Dialysis/
21. dialysis.tw
22. ((end-stage renal disease) or (end-stage kidney disease)).tw
23. Or/19-22
24. 18 and 23

Search strategy for registered trials in the WHO International Clinical Trials Registry Platform

1. Dialysis
2. H(a)emodialysis
3. End-stage renal disease (end-stage kidney disease)

Search strategy for funded clinical research studies in national funding agencies and kidney research funding organisations of Australia, Canada, U.K., and U.S.

1. H(a)emodialysis
2. Peritoneal dialysis
3. End-stage renal disease (end-stage kidney disease)
