# Supplementary material for: Assessing the extent to which current clinical research is consistent with patient priorities: a scoping review using a case study in patients on or nearing dialysis
Source: Can J Kidney Health Dis. 2015 Oct 1;2:35. doi: 10.1186/s40697-015-0070-9 (PMC4590701; doi:10.1186/s40697-015-0070-9)
Supplement: Additional file 2: — The distribution of the proportion of studies which were consistent with at least 1 of the top 10 dialysis patient priorities by year (2010 – 2013). (DOCX 81 kb) [file 40697_2015_70_MOESM2_ESM.docx]

**Additional file 2**


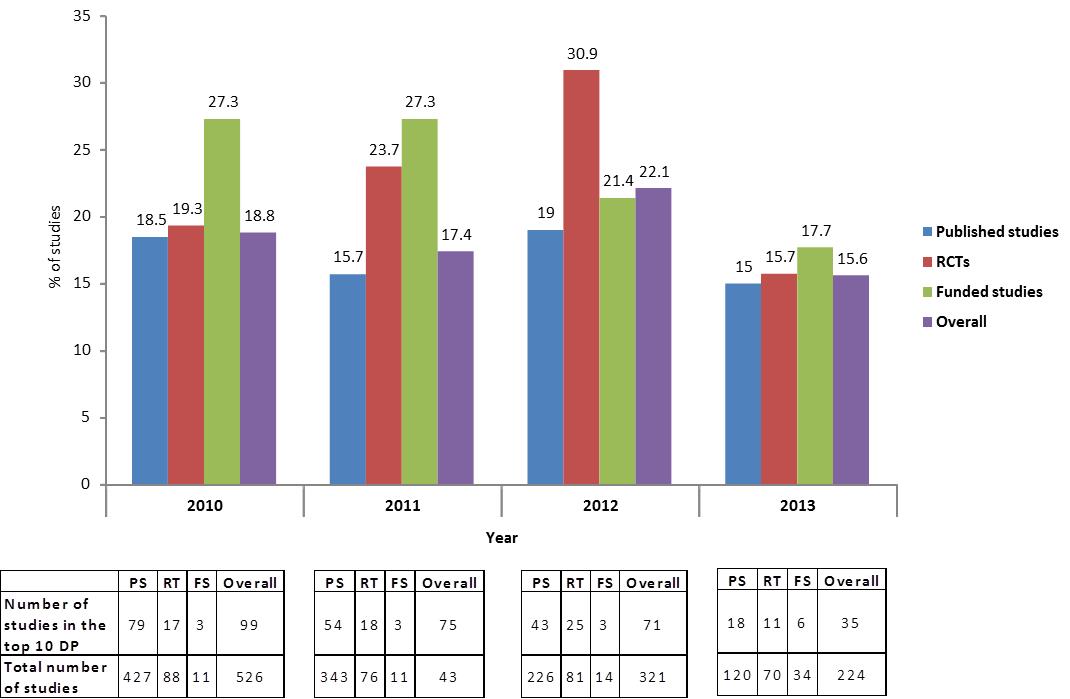


The distribution of the proportion of studies which were consistent with at least 1 of the top 10 dialysis patient priorities by year (2010 – 2013)
