# Supplementary material for: Assessing the extent to which current clinical research is consistent with patient priorities: a scoping review using a case study in patients on or nearing dialysis
Source: Can J Kidney Health Dis. 2015 Oct 1;2:35. doi: 10.1186/s40697-015-0070-9 (PMC4590701; doi:10.1186/s40697-015-0070-9)
Supplement: Additional file 3: — Studies not addressing the top 10 research priorities categorized based on a 41 category taxonomy of major kidney research areas. (DOCX 69 kb) [file 40697_2015_70_MOESM3_ESM.docx]

**Additional file 3**


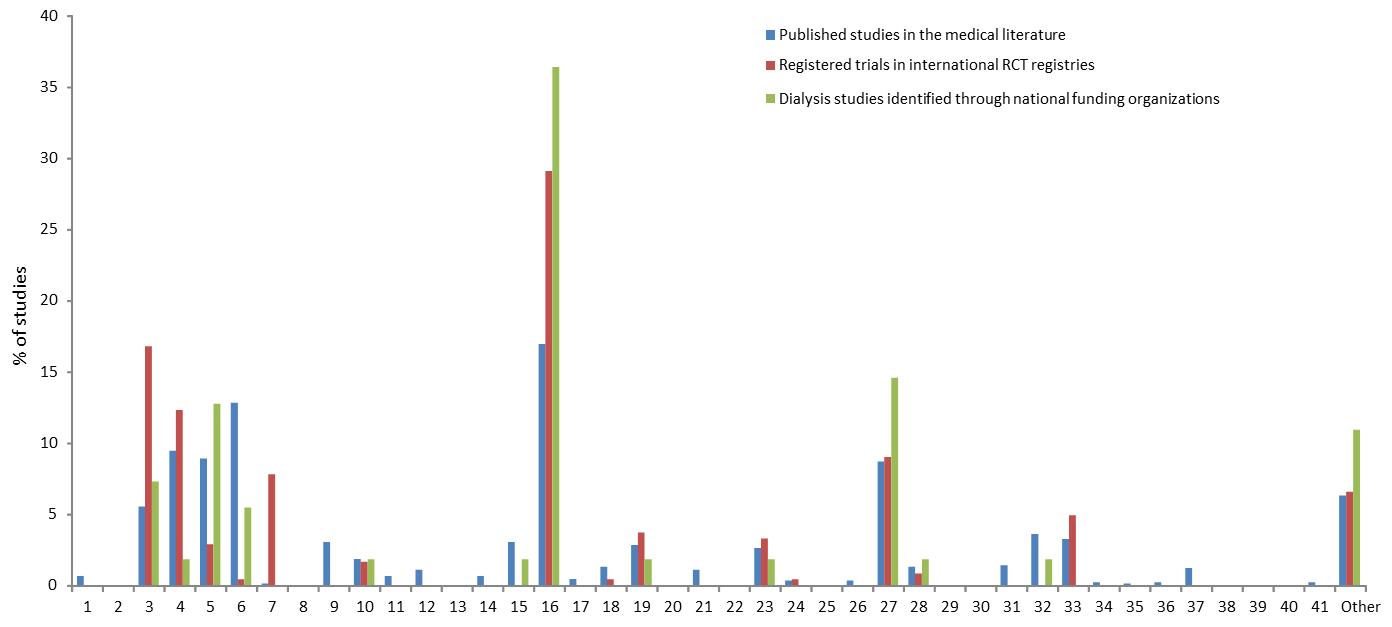


Additional file 3: Studies not addressing the top-10 research priorities categorised based on a 41-category taxonomy of major kidney research areas; (1) Acid-base, fluid, and electrolytes; (2) Acute kidney injury/acute renal failure;* (3) Anemia, iron, erythropoietin; (4) Bone and mineral metabolism; (5) Cardiovascular disease^; (6) End-stage renal disease (epidemiological studies unrelated to the top-10 priorities); (7) Continuous dialysis therapies (including continuous ambulatory peritoneal dialysis); (8) Cystic diseases of the kidney; (9) Health economics; (10) Endocrinology and diabetes; (11) Ethics (including end-of-life care); (12) Gastroenterology and hepatology; (13) Glomerular filtration (including assessment/estimation methods of GFR); (14) Glomerulonephritides; (15) Healthcare disparities; (16) Haemodialysis^; (17) Hereditary kidney diseases; (18) Hypertension^; (19) Infectious disease; (20) Medical education; (21) Methodology (including biostatistics and clinical epidemiological methods); (22) Nephrolithiasis; (23) Nutrition^; (24) Oncology; (25) Paraneoplastic and monoclonal disorders; (26) Pathology; (27) Peritoneal dialysis^; (28) Pharmacology; (29) Pregnancy-associated kidney diseases; (30) Proteinuria; (31) Quality improvement; (32) Quality of life^; (33) Radiology/interventional nephrology; (34) Renal artery disease; (35) Rheumatology; (36) Toxicology and exposures; (37) Transplantation^; (38) Tubulointerstitial disease; (39) Urinalysis; (40) Urology; (41) Vasculitides and associated conditions

*the research taxonomy we used to categorise studies not addressing the top-10 research priorities included acute kidney injury (AKI) as a major research area – our review excluded studies in AKI

^7 of the 41 categories (“Cardiovascular disease”, “Haemodialysis”, “Hypertension”, “Nutrition”, “Peritoneal dialysis”, “Quality of life”, and “Transplantation”) included broad areas of potential relevance to the top-10 research priorities, although not all studies in these 7 categories addressed the top-10 priority areas (further details in Methods)
